# The histone deacetylase inhibitor, LBH589, promotes the systemic cytokine and effector responses of adoptively transferred CD8+ T cells

**DOI:** 10.1186/2051-1426-2-8

**Published:** 2014-04-15

**Authors:** Dominique N Lisiero, Horacio Soto, Richard G Everson, Linda M Liau, Robert M Prins

**Affiliations:** 1 Department of Molecular and Medical Pharmacology, 650 Charles E. Young Drive South, 23-120 Center for Health Sciences, Los Angeles, CA 90095-1735, USA; 2 Department of Neurosurgery, University of California-Los Angeles School of Medicine, Center for Health Sciences, Room 74-145 CHS, 10833 Le Conte Avenue, Box 956901, Los Angeles, CA 90095-6901, USA; 3 Jonsson Comprehensive Cancer Center, University of California–Los Angeles School of Medicine, 8-684 Factor Building, Box 951781, Los Angeles, CA 90095-1781, USA; 4 Brain Research Institute, University of California-Los Angeles School of Medicine, 695 Charles E. Young Drive South, Los Angeles, CA 90095, USA

**Keywords:** T cells, Tumor immunity, Dendritic cells, Inflammation

## Abstract

**Background:**

Histone deacetylase (HDAC) inhibitors are a class of agents that have potent antitumor activity with a reported ability to upregulate MHC and costimulatory molecule expression. We hypothesized that epigenetic pharmacological immunomodulation could sensitize tumors to immune mediated cell death with an adoptive T cell therapy.

**Methods:**

The pan-HDAC inhibitor, LBH589, was combined with gp100 specific T cell immunotherapy in an *in vivo* B16 melanoma model and in an *in vivo* non-tumor bearing model. Tumor regression, tumor specific T cell function and phenotype, and serum cytokine levels were evaluated.

**Results:**

Addition of LBH589 to an adoptive cell transfer therapy significantly decreased tumor burden while sustaining systemic pro-inflammatory levels. Furthermore, LBH589 was able to enhance gp100 specific T cell survival and significantly decrease T regulatory cell populations systemically and intratumorally. Even in the absence of tumor, LBH589 was able to enhance the proliferation, retention, and polyfunctional status of tumor specific T cells, suggesting its effects were T cell specific. In addition, LBH589 induced significantly higher levels of the IL-2 receptor (CD25) and the co-stimulatory molecule OX-40 in T cells.

**Conclusion:**

These results demonstrate that immunomodulation of adoptively transferred T cells by LBH589 provides a novel mechanism to increase *in vivo* antitumor efficacy of effector CD8 T cells.

## Background

The adoptive transfer of tumor specific T cells is becoming a viable treatment option for many patients with solid tumors [1]. However, despite the ability to specifically target tumors, adoptively transferred cells often fail to survive and persist *in vivo*[2]. Their ability to retain effector function diminishes quickly and often transforms into a suppressive state, with an inability to mobilize IFN-γ and other critical cellular functions [3-5]. Many strategies have been employed to reverse this balance in favor of an effector T cell response. Strategies such as lymphodepletion prior to adoptive cell transfer and high doses of IL-2 have been shown to significantly increase retention and proliferation of adoptively transferred T cells [6,7]. In addition, combining these strategies with immunomodulation of the tumor itself has shown the potential to increase responsiveness to immunotherapy [8].

Histone deacetylase inhibitors (HDACis), such as vorinostat (SAHA) and romidepsin, are approved for the treatment of cutaneous T-cell lymphomas. Such agents act via multiple mechanisms, including cell cycle arrest and activation of the intrinsic death pathway [9,10]. Acetylation and deacetylation of lysine residues on histone tails and non-histone substrates controls a number of cellular processes including the regulation of transcription, transcription factor stability and cell survival. Histone deacetylases are a class of enzymes responsible for deacetylation of histone proteins and other non-histone protein substrates [11]. Inhibition of histone deacetylases with HDACis, such as vorinostat, can preferentially induce cell cycle arrest, apoptosis, and differentiation in leukemic malignancies and solid tumors [12]. More importantly, HDACis have been shown to enhance tumor immunity by upregulating the expression of major histocompatibility class (MHC) molecules, costimulatory molecules and components involved in tumor necrosis factor (TNF) superfamily signaling [13-17]. However, HDACi’s have also been shown to increase the function of T regulatory cells and increase IDO immunosuppression by dendritic cells [18-20]. These potentially contradictory functions of HDACis in the context of tumor immunity have complicated their inclusion in immunotherapy protocols.

In order to test whether an HDACi could synergize with immunotherapy in an *in vivo* melanoma model, we utilized LBH589 (Panobinostat) in combination with T cell transfer therapy. LBH589 is a cinnamic hydroxamic acid derivative with broad inhibitory activity of class I, II, and IV HDACs in the low nanomolar range [21]. It has shown clinical efficacy for the treatment of multiple myeloma and Hodgkin’s lymphoma and animal models in doses ranging from 10-100 mg/kg [22,23]. However, whether LBH589 could similarly enhance adoptive T cell transfer without generating a potentially immunosuppressive milieu had yet to be addressed. We utilized gp100 tumor associated antigen specific Pmel T cell immunotherapy in an *in vivo* melanoma model in order to address these concerns. Adjuvant administration of LBH589 potently synergized with adoptive cell transfer, and to our surprise, created a highly pro-inflammatory environment that could be measured by significant modulation of serum cytokine levels. This was accompanied by a significant expansion and enhancement of effector function, which occurred in the presence or absence of tumor. Notably, specific release of TNF following restimulation of Pmel T cells and serum cytokine levels of TNF were significantly increased and sustained over time. Taken together with an increase in the T cell specific expression of the TNF superfamily receptor, OX-40, inclusion of LBH589 highlights the potential new role of HDAC inhibitors in modulating and sustaining *in vivo* T cell function.

## Results

### LBH589 synergizes with an adoptive cell transfer therapy to reduce tumor burden

**Figure 1:**
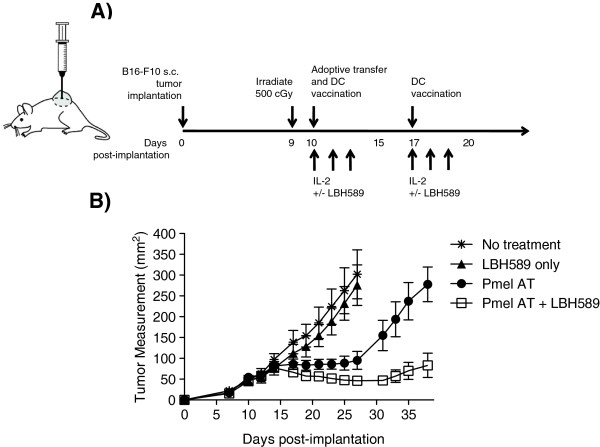
**LBH589 in combination with an adoptive cell transfer therapy reduces tumor burden in a subcutaneous B16-F10 melanoma model. A**. Treatment schedule. **B**. Tumor growth measurements of the mean area of tumors in control mice treated with irradiation only, LBH589 following irradiation, Pmel T cell adoptive transfer, or Pmel T cell adoptive transfer therapy with LBH589 (n = 8 mice/group). Results are representative of at least three independent experiments. Two-way ANOVA analyses were performed to determine statistical significance between treatment groups.

Significant controversy exists about whether HDACi tolerize or enhance anti-tumor immune responses. In addition, the mechanisms by which HDACi alter immune responsiveness are not well understood. We previously reported that another HDACi similar to LBH589 (LAQ824) could enhance ACT in a mouse model [24]. However, it was unclear mechanistically how a pan-HDAC inhibitor might synergize with adoptively transferred, tumor-specific T cells *in vivo*. To test this new agent, mice with 10 day established B16 tumors were lymphodepleted with 500 cGy irradiation one day prior to adoptive transfer. Treated groups then received 5 × 10^6^ gp100-specific Pmel T cells, supported by a gp100_25-33_ peptide pulsed dendritic cell vaccination, and systemic IL-2 with or without LBH589 (5 mg/kg). One week later, this was followed by a second DC vaccination and IL-2 (Figure 1A). As a single agent, LBH589 (5 mg/kg) did not decrease tumor burden in comparison to untreated groups leading us to conclude that this was an appropriate subtherapeutic dose (p = 0.45). However, LBH589 in combination with Pmel adoptive transfer therapy, showed significantly greater control of tumor growth compared with Pmel ACT alone (p = 0.019) (Figure 1B). Thus, LBH589 was able to dramatically enhance the efficacy of a T cell based therapy.

### LBH589 alters and extends in vivo peripheral cytokine production

**Figure 2:**
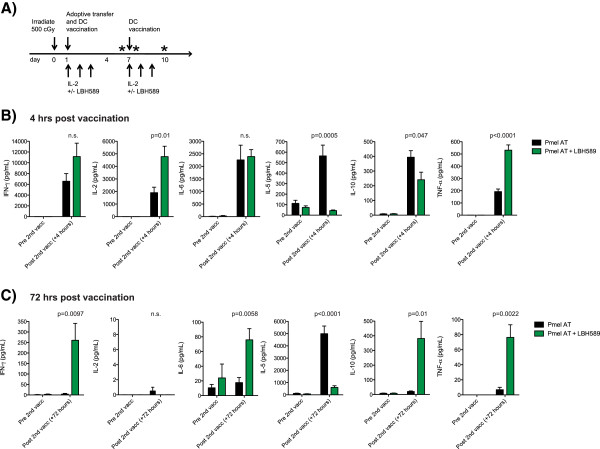
**Treatment with LBH589 alters the peripheral cytokine milieu of non-tumor bearing mice treated with LBH589. A**. Treatment scheme. Asterisks indicate timepoints in which serum was collected. **B**. Serum levels of cytokines in groups treated with and without LBH589. Serum was taken one day prior to dendritic cell vaccination, and then four hours post vaccination. **C**. Serum cytokine levels of the same treatment groups taken prior to dendritic cell revaccination on day 7 and then 72 hours following revaccination. Serum from at least 6 mice was measured per cytokine. Student’s t-tests were performed to determine statistical significance between groups.

Widely varied immunomodulatory effects of HDACi have been reported in a number of different models [16,23,25]. The majority of these studies have highlighted the anti-inflammatory properties of HDACi’s. In particular, it is known that HDACi’s can dampen the anti-tumor immune response, and reportedly involve the functional enhancement and generation of T regulatory cells and the production of IDO by dendritic cells. However, given the therapeutic benefit observed in this *in vivo* tumor model, we hypothesized that the administration of an HDACi after the induction of lymphopenia and adoptive cell transfer might alter the dynamics of the systemic immune response differently. In order to assess global changes in the inflammatory environment, we quantified peripheral blood serum cytokine levels at 3 distinct time points following T cell ACT and DC vaccination with, and without, LBH589 administration (Figure 2A). The first sample was obtained one hour prior to DC vaccination. The second and third serum samples were then obtained 4 hours and 72 hours following vaccination respectively. A dramatic shift in the TH1 and pro-inflammatory cytokine production was observed 4 hours following DC vaccination (Figure 2B). This shift was highlighted by a significant release of TNF and IL-2, and a significant reduction in IL-5 and IL-10 in groups treated with LBH589 and adoptive transfer compared with groups that only received Pmel adoptive transfer. Furthermore, these significant shifts in pro-inflammatory cytokine production were still noticeable 72 hours following vaccination. Notably, serum levels of IFN-γ ,TNF, and IL-10 were significantly elevated in mice treated with LBH589 and adoptive transfer. These results are impressive considering the serum half-life of TNF is approximately 10 minutes [26]. Furthermore, the potency of this inflammatory response 72 hours following vaccination is exemplified by an increase in serum IL-10. Although we were unable to determine the source of this IL-10 due to technical limitations, we hypothesize that highly activated Pmel T cells utilized this as a mechanism to control immunopathology. These surprising results point towards a prolonged and sustained global shift towards a pro-inflammatory environment *in vivo*.

### Expansion of adoptively transferred cells and reduction of T regulatory cells with LBH589 treatment

**Figure 3:**
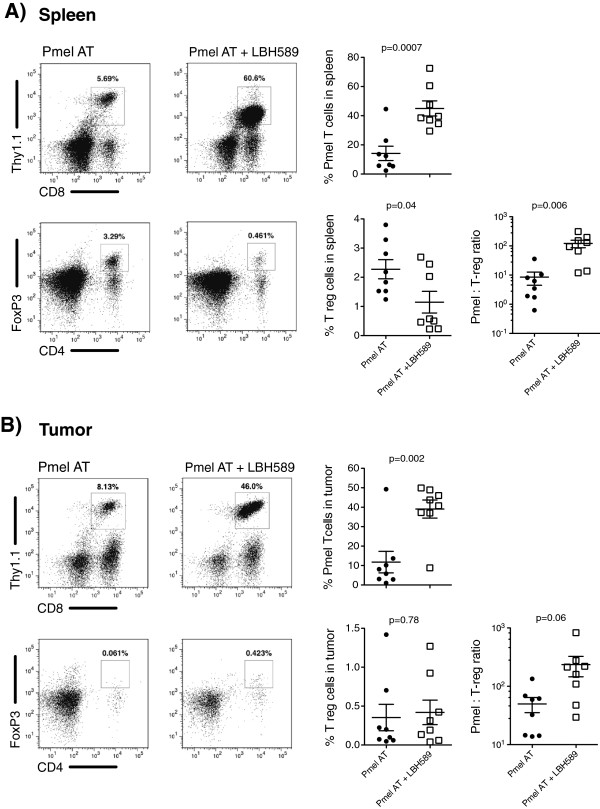
**Dramatic expansion and recovery of tumor specific T cells *in vivo *as a result of LBH589 treatment. A**. Quantification of Pmel T cells recovered from splenocytes collected 10 days after adoptive cell transfer according to the treatment scheme on Figure 1A. Representative flow cytometry plots shown are derived from a lymphocyte gate followed by a live cell gate. Pmel: T reg ratios are determined by utilizing absolute numbers of Pmel T cells (CD8+ Thy1.1+) to the absolute number of T reg cells (CD4+ CD25hi FoxP3+). **B**. Quantification of lymphocytes within the tumor. Representative flow cytometry plots of lymphocytes isolated from subcutaneous B16 melanomas digested with collagenase 10 days following adoptive cell transfer. Dot plots shown are derived from lymphocyte and live cell gates. The Pmel: T reg ratio was calculated using absolute number of Pmel T cells and T reg cells per mg of tumor. A and B *dark circles* indicate mice treated with adoptive cell transfer and *open squares* indicate mice treated with adoptive cell transfer with LBH589 and each symbol represents one mouse. Each experiment described has been conducted two times with similar results and included at least four mice per group. Student’s t-tests were performed to determine statistical significance between groups.

The previously reported anti-inflammatory properties of HDAC inhibitors have generated little enthusiasm for their concurrent use in cancer immunotherapeutic strategies. However, with such extensive changes observed in the cytokine environment, we investigated whether the co-administration of LBH589 influenced the expansion of Pmel T cells and other endogenous lymphocyte populations. We quantified the percentages and absolute numbers of adoptively transferred Pmel T cells and endogenous regulatory T cell populations in mice with established B16-F10 tumors (Figure 3A) We were surprised to find that there was a significant increase in the percentage of Pmel T cells (CD8+ Thy1.1+) recovered from the spleen 3 days following DC vaccination (45% CD8 + Thy1.1+ with LBH589 vs. 14% for placebo control; p = 0.0007) (Figure 3A). In contrast to what has been reported, this expansion of adoptively transferred T cells was accompanied by a decrease in both the percentage and absolute number of endogenous T regulatory cells (CD3+ CD4+ FoxP3+) recovered in the spleen from 2.1% to 1.1% in mice treated with LBH589 (p = 0.039) (Figure 3A). This significantly altered the tumor specific T cell to T regulatory cell ratio in the periphery in mice treated with and without LBH589 respectively (121.9 vs. 8.1; p = 0.0064). Furthermore, this was accompanied by a greater percentage of Pmel T cells within the tumor when groups were treated with LBH589 than treated without (39% vs. 11.7% respectively; p = 0.0021), and an increased, though not significant, density of tumor-infiltating Pmel T cells (Figure 3B). Although the T regulatory cell frequency was low inside these the tumors, the overwhelming tumor infiltrating lymphocyte population enhanced the Pmel to T reg cell ratio to 235.5 from 49.7 for mice treated with and without LBH589, respectively (p = 0.06). This data suggests that the result of HDACi coadministration can provide a pro-inflammatory environment during an antitumor response in lymphodepleted hosts that serves to support the expansion and survival of tumor-specific T cells following adoptive transfer.

### Enhanced T cell expansion with LBH589 is not dependent on the presence of tumor

**Figure 4:**
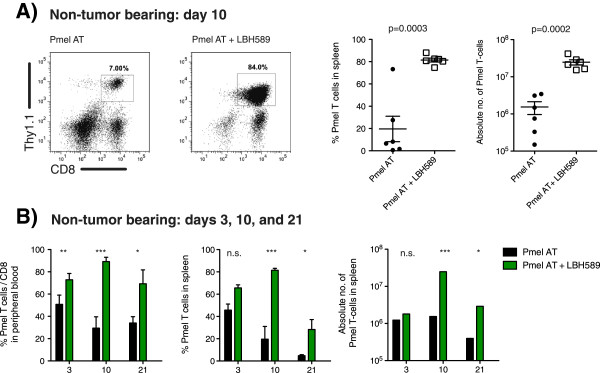
**Recovery and systemic expansion of Pmel T cells with LBH589 also occurs in the absence of tumor. A**. Expansion of Pmel T cells treated by the same scheme as shown in Figure 1A, but in non-tumor bearing irradiated hosts. Representative dot plots indicate the percentage and absolute number of splenocytes that are Pmel T cells on day 10 following adoptive cell transfer. **B**. Analysis of Pmel T cell expansion in both peripheral blood and spleen over time. Pmel T cell expansion in peripheral blood is expressed as the percentage of Pmel T cells in the overall CD8 T cell population days 3, 10, and 21 days following adoptive cell transfer. Results described are representative of at least three experiments with similar results. Student’s t-tests were performed to determine statistical significance between groups.

The ability of particular HDACi to immunosensitize tumors has been documented and could explain the observed synergism between ACT immunotherapy and LBH589 [13-16]. However, whether LBH589 directly influenced T cell proliferation in the absence of tumor was still unknown. In order to test whether LBH589 mediated its effects independently from its effects on the tumor, we adoptively transferred 5 × 10^6^ Pmel T cells into lymphodepleted, non-tumor bearing hosts. These cells were again supported by a peptide pulsed DC vaccination and IL-2, with or without LBH589. Ten days following adoptive transfer, and 3 days following vaccination, Pmel T cells constituted 81% of the splenic cellularity (2.5 × 10^7^ cells) in groups receiving LBH589, while Pmel T cells only represented 19% (1.5 × 10^6^ cells) without LBH589 (p < 0.001) (Figure 4A). Furthermore, this expansion was not limited to the spleen, but was also observed in the percentage of Pmel T cells in the overall CD8 population from peripheral blood (Figure 4B). This expansion was the most dramatic following vaccination. In addition to enhanced expansion, retention of Pmel cells also persisted 21 days following adoptive transfer (11 days post vaccination). Thus the, combination of an HDACi with a T cell based immunotherapy was not immunosuppressive. Instead, the coadministration of LBH589 resulted in increased retention of adoptively transferred T cells.

### Increased ex vivo polyfunctionality of tumor specific T cells with LBH589

**Figure 5:**
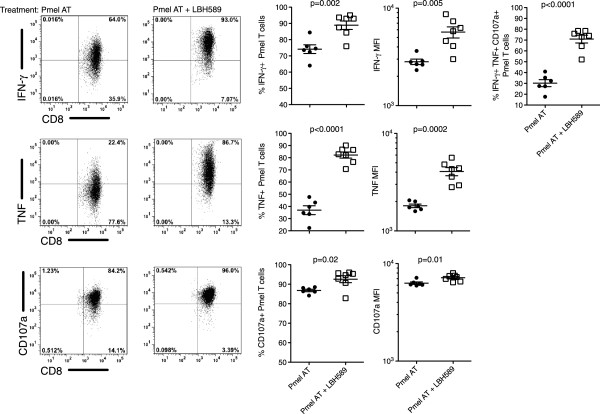
**Functional enhancement of Pmel T cell cytokine production.** Restimulation of Pmel T cell splenocytes with cognate gp100_25-33_ peptide *ex vivo* for five hours. Representative plots indicate splenocytes restimulated 10 days following adoptive cell transfer and are gated from CD8+ Thy1.1+ Pmel T cells. Gates were set based on Pmel cells that lacked peptide stimulation for the 5 hour duration of the restimulation. CD107a staining, as a marker for degranulation, was achieved by addition of CD107a antibody just immediately prior to the restimulation period. Results described are representative of at least three experiments with similar results. Student’s t-tests were performed to determine statistical significance between groups.

Realizing that LBH589 mediated such a profound effect on the retention of adoptively transferred Pmel T cells, we also addressed whether the function of these cells was likewise enhanced. Optimal expansion of Pmel T cells occurred in the days following revaccination, providing an ideal timeframe for us to assess their ability to degranulate (CD107a+/LAMP-1+) and/or secrete the cytokines IFN-γ and TNF. Lymphodepleted, non-tumor bearing mice were adoptively transferred with 5 × 10^6^ Pmel T cells and supported with a dendritic cell vaccine and IL-2 with or without LBH589. Ten days following adoptive transfer, splenocytes from mice were obtained and restimulated with gp100_25-33_ peptide for five hours. During this time, the expression of CD107a was measured in order to quantify the percentage of T cells undergoing degranulation. In groups treated with LBH589, we observed significant increases in Pmel T cell cytokine secretion and degranulation. The percentage and MFI of Pmel T cells secreting IFN-γ was significantly increased in mice treated with LBH589 compared with those treated without (88.9% vs. 74.1% respectively, p < 0.005) (Figure 5). Furthermore, the percentage of Pmel T cells exhibiting mobilization of the degranulation marker, CD107a, was also increased in mice treated with LBH589 compared with those treated without (92.5% vs 86.8% respectively, p < 0.05) (Figure 5). The most dramatic difference observed for LBH589 treated groups was more than a 2-fold increase in the percentage of cells secreting TNF in comparison to those not treated (82.2% vs. 36.9% respectively, p < 0.001) (Figure 5). When considering the number of cells able to secrete both cytokines and demonstrate the ability to degranulate, more than 70.9% of Pmel cells simultaneously showed polyfunctionality when treated with LBH589 in comparison to 30.2% in groups not treated with LBH589 (p < 0.0001) (Figure 5). Furthermore, this is in stark contrast to three days following adoptive transfer when only 10% of untreated and 20% of LBH589 treated Pmel T cells were able to secrete TNF (Additional file 1: Figure S1). This demonstrated a significant enhancement in the ability to respond to a peptide specific vaccination with elevated TNF secretion. For the first time, these results demonstrate the ability of an HDAC inhibitor to increase the *in vivo* polyfunctional capacity of adoptively transferred CD8 T cells *in vivo*.

### LBH589 enhances markers of activation on tumor specific T cells

**Figure 6:**
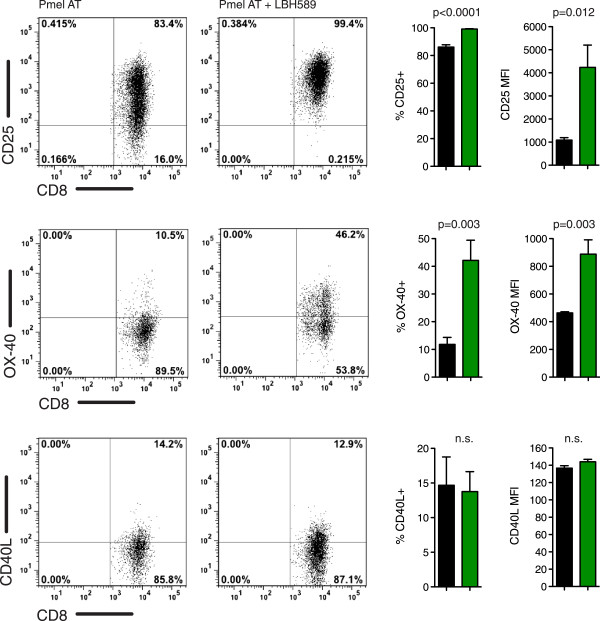
**LBH589 increases expression of markers of T-cell activation and costimulation.** Splenocytes from mice treated with and without LBH589 were assayed for the expression of CD25, OX-40, CD40L and ten days after adoptive cell transfer. Representative plots are gated on CD8+ Thy1.1+ Pmel T cells. Each group contained 6 mice with similar data obtained in an independent experiment. Student’s t-tests were performed to determine statistical significance between groups.

The TNF superfamily of ligands and receptors, as well as TNF itself, are known to be modulated by HDACi’s in numerous cancer cell lines [13,27-29]. Because our prior results demonstrated significant enhancement of TNF secretion by restimulated Pmel T cells, we investigated whether LBH589 might influence the expression of the TNF superfamily of ligands and receptors, as well as other markers of T cell activation. We showed previously that the high affinity IL-2 receptor α chain (CD25) was important for proliferative and *in vivo* antitumor activity of CD8 T cells [30]. In order to assess whether this was also the case in mice treated with LBH589, we recovered splenocytes from non-tumor bearing mice 10 days following adoptive cell transfer. Pmel T cells recovered from groups treated with LBH589 showed a significantly increased percentage of positively staining cells and MFI of CD25 expression (Figure 6). At this time, we also examined TNF family members normally expressed on activated CD8 T cells. No significant changes in the expression of CD40L (Figure 6), 4-1BB, or CD27 were detected (data not shown). However, OX-40 was significantly upregulated on Pmel T cells following vaccination in groups treated with LBH589. These results demonstrate that utilization of LBH589 promotes a pro-inflammatory environment and alters the T cell effector phenotype through the expression of CD25 and OX-40.

## Discussion

Our study addressed whether the adjunctive use of an HDAC inhibitor could synergize with an adoptive cell transfer therapy, without suppressing or compromising a tumor specific effector T cell response. Utilization of LBH589 dramatically enhanced the antitumor activity of an adoptive T cell transfer therapy in a murine B16-F10 subcutaneous melanoma model. Inclusion of LBH589 together with adoptive T cell transfer induced significant regression of established B16 melanoma tumors while generating a systemic proinflammatory cytokine milieu, illustrated by the sustained release of IFN-γ and TNF. Such enhanced antitumor activity was further exemplified by the increased recovery of adoptively transferred Pmel cells, both systemically and intratumorally, together with a drastic reduction in the T regulatory cell population. Surprisingly, enhanced Pmel T cell *in vivo* expansion and effector function occurred even in the absence of tumor in LBH589 treated groups. Phenotypically, following adoptive transfer and treatment with LBH589, Pmel T cells preferentially expressed high levels of the TNF receptor family member, OX-40, and secreted high levels of TNF following *ex vivo* restimulation. These results highlight the significant antitumor and immunomodulatory activities of LBH589.

Our findings demonstrated that the adjunctive use of LBH589 with adoptive T cell transfer significantly reduces T regulatory cell populations in the periphery and within the tumor. This is significant when considering the immunomodulatory properties of T reg cells in the context of a tumor mass. Pre-clinical studies have demonstrated that the infiltration of T reg cells specifically within a tumor mass decreases effector T cell proliferation and function [31]. Furthermore, pharmacological blockade of a known T reg cell marker, CTLA-4, restored effector T cell proliferation and synergized with a therapeutic vaccine. In fact, the ability to modulate T regulatory cell populations *in vivo* and significantly elevate the T effector to T regulatory cell ratio can act as a positive predictive factor for tumor rejection and promotes effector cell proliferation and cytokine secretion [32]. Clinically, high levels of peripheral T regulatory cell populations are negatively associated with therapeutic responsiveness after lymphodepletion and adoptive cell transfer in patients [33]. Although this T regulatory cell population may not be indicative of a tumor educated population, it may highlight the overall status of immune responsiveness. Our results, as well as evidence from both pre-clinical and clinical studies, highlight the significance of T regulatory cell function in the context of immune based therapies. Thus, the selective pharmacological modulation of T effector function and T regulatory cell populations to significantly increase anti-tumor activity makes LBH589 an extremely attractive therapeutic that may abrogate the need for lymphodepletion prior to adoptive cell transfer. Future studies are needed to address this hypothesis.

Our results also indicated an enhanced inflammatory cytokine environment and state of T cell responsiveness. In this light, we tested the ability of LBH589 to modulate T cell function in non-tumor bearing hosts. We determined that the majority of immunological effects seen in our treatment regimen were due to an overwhelming enhancement of T cell proliferation and function by LBH589, even in the absence of tumor. Although one of the limitations of our experimental design was that it did not allow us to specifically distinguish whether LBH589 acts directly on adoptively transferred T cells, dendritic cells, or endogenous lymphocytes we hypothesize that in this lymphopenic environment, the majority of effects are on the activated T cells themselves. This is supported by the fact that the lymphocytes in the spleen of LBH589 treated groups were composed primarily of Pmel CD8+ T cells, and a significantly lower percentage of CD4+ T cells and CD4+ T regulatory cells. The enhancement of T cell function we observed was dominated by a more than 2 fold change in the tumor peptide (gp100_25-33_) specific secretion of TNF *ex vivo*, mirroring the data observed in serum cytokine levels. This is the first study that demonstrates a pharmacological enhancement of polyfunctional T cell status by an HDAC inhibitor. The potency of this response was magnified if a third dendritic cell vaccination was utilized at day 14 of this treatment scheme. This resulted in a lethal cytokine storm in a majority of mice treated with immunotherapy and LBH589. From this data we concluded that only one dendritic cell revaccination was necessary to mount an effective anti-tumor response without adverse events. This warrants further investigation into the molecular mechanisms for HDACi mediated regulation of inflammatory cytokines, and whether sub-optimal doses of IL-2 can be utilized to support adoptively transferred cells. These results are also surprising considering previous studies have shown that LBH589 impairs the function and phenotype of dendritic cells by downregulating co-stimulatory molecules and repressing inflammatory cytokine production [34]. Our own studies, which utilize a peptide specific dendritic cell vaccine in combination with adoptive T cell transfer, demonstrated robust tumor specific T cell specific proliferation and cytokine production following dendritic cell revaccination. Future studies will investigate whether LBH589 modulates dendritic cell function in the therapeutic context of adoptive T cell transfers.

HDAC inhibitors have been shown to preferentially regulate the expression of TNF superfamily members, including TNF receptors, and TNF associated ligands in tumors. The class I HDAC inhibitor, depsipeptide increased the expression of TNF-related apoptosis-inducing ligand (TRAIL) on chronic lymphocytic leukemia (CLL) and acute lymphoblastic leukemia cell lines, sensitizing them to death receptor-induced apoptosis [16]. Additionally, inhibition of HDAC11 in Hodgkin lymphoma increases the expression of OX-40 ligand and inhibits the generation of IL-10 producing T regulatory cells *in vitro*[29]. However, the modulation of TNF family members by HDAC inhibitors in CD8+ T cells has not been previously demonstrated. In this study, we demonstrated an increase in the specific expression of TNF and TNF superfamily member, OX-40, by tumor-specific T cells. TNF has been shown to be crucial for the priming and effector function of CD8 T cells during an antitumor immune response [35,36]. This is especially significant considering that proper costimulation and cytokine support for effective T cell responses is severely lacking during tumor surveillance. OX-40 is a critical co-stimulatory molecule that is necessary for effector function, survival, and memory generation [35,37]. *In vivo*, OX-40 agonistic antibodies potentiate CD8 T cell memory generation and antitumor activity [38-40]. Therefore, inclusion of LBH589 in current adoptive cell transfer protocols could potentially enhance the anti-tumor activity of T cells by providing *in vivo* co-stimulation through OX-40. Taken together with the increased expression of the high affinity IL-2 receptor, CD25, we believe that increased expression of OX-40 imparts a highly activated phenotype to CD8+ T cells that allows them to compete for proliferative cytokines (IL-2) and co-stimulation through OX-40. We believe that LBH589 may specifically modulate the proliferation, retention, and responsiveness of CD8 T cells.

We have demonstrated that LBH589 potentiates the function of CD8 T cells, and this occurs in the presence and absence of tumor. We demonstrated that an HDAC inhibitor has the potential to sensitize tumor specific cells to peptide specific vaccination and acquisition of full T cell effector function, by increasing peptide specific secretion of TNF, expression of the co-stimulatory receptor, OX-40, and expression of the IL-2 high affinity receptor, CD25. Further investigations need to be conducted in order to determine whether LBH589 directly modulates histone acetylation in CD8+ T cells and transcriptionally regulates T cell effector function and expression of TNF superfamily members. Furthermore, although the HDAC target(s) of LBH589 inhibition responsible for these immunomodulatory activities is unclear, this study provides additional mechanistic insight into the ability of HDACs to specifically regulate T cell function and anti-tumor activity. Our results highlight LBH589 as a safe and effective adjuvant to regulate the proliferation and function of adoptively transferred tumor specific T cells, and potentially warrants its inclusion in future ACT human clinical trials.

## Conclusions

The histone deacetylase inhibitor, LBH589, synergizes with adoptive T cell transfer therapy and helps mediate potent antitumor activity in an *in vivo* melanoma model. In this immunotherapeutic context, LBH589 enhanced the retention of polyfunctional tumor specific T cells, promoted systemic cytokine responses, and increased the effector to regulatory T cell ratio. These results demonstrate the ability of a pan histone deacetylase inhibitor to effectively modulate the antitumor response and warrants further studies for future clinical use.

## Methods

### Animals and cell lines

All mice were bred and kept under defined-flora pathogen-free conditions at the Association for Assessment and Accreditation of Laboratory Animal Care-approved animal facility of the Division of Experimental Radiation Oncology at the University of California Los Angeles. Mice were handled in accordance with the University of California Los Angeles animal care policy and approved animal protocols. The B16-F10 murine melanoma cell line was obtained from American Type Culture Collection (Rockville, MD).

### Tumor implantation and lymphodepletion

For studies analyzing tumor growth over time in an *in vivo* melanoma model, C57BL/6 mice (6-12 weeks of age) were implanted subcutaneously in the lower left flank with 2 × 10^5^ B16-F10 melanoma cells and allowed to establish for 10 days. For studies analyzing tumor infiltrating lymphocytes, 2.5 × 10^5^ B16-F10 melanoma cells were implanted in the same location and allowed to establish for 10 days.

One day prior to adoptive T-cell transfer, lymphopenia was induced by 500 cGy total body irradiation.

### Adoptive T cell transfer and dendritic cell vaccination

Pmel-1 T cells and bone marrow derived dendritic cells were generated as previously described [30]. In brief, naïve Pmel-1 splenocytes were activated with human gp100_25-33_ peptide (NH_2_-KVPRNQDWL-OH, 1 *u*g/ml; Biosynthesis, Lewisville, TX) and 100 IU/ml human IL-2 (National Cancer Institute Preclinical Repository, Developmental Therapeutics Program) for 72 hours [30]. These cells were then re-cultured for an additional 48 hours in 100 IU/ml IL-2 without hgp100. Pmel-1 T-cells (5 × 10^6^) were injected i.v. in 0.1 ml PBS.

Bone marrow derived dendritic cells were generated as previously described [30]. Bone marrow cells from the femurs and tibias of 2 Bl/6 mice were initially cultured overnight in a petri dish in RPMI 1640 supplemented with 10% FBS and pencillin/streptomycin. The next day, non-adherent cells were collected and washed twice with media and re-cultured in 50 ml of fresh media containing 2 ng/ml recombinant murine GM-CSF and 10 ng/ml recombinant murine IL-4 (Peprotech). Cells were then plated at 1 ml per well in 24 well plates and cultured for 3 days. On the third day, 0.5 ml of media was removed and 1 ml of new media containing 10 ng/ml IL-4 and 2 ng/ml GM-CSF was added. On the 7^th^ day, cells were harvested by using a syringe plunger to scrape cells from the bottom of each well. Once harvested, dendritic cells were pulsed with human gp100_25–33_ peptide at a concentration of 10 μM for 90 min at room temperature. Approximately 5 × 10^5^ were injected subcutaneously at four sites on the back [41]. IL-2 (5 × 10^5^ IU) was administered in 500 ul and given as an intraperitoneal injection. LBH589 (a kind gift from Novartis) was given at a dose of 5 mg/kg and administered at the same time as IL-2.

### Ex vivo Pmel-1 T cell stimulation and intracellular FACS staining

After the indicated time periods following adoptive T cell transfer, splenocytes were enumerated and restimulated with or without hgp100_25-33_ peptide. GolgiPlug protein transport inhibitor (BD Biosciences) and allophycocyanin-conjugated anti-CD107a mAb (2 ug, clone 1D4B; BD Biosciences) were added to each well containing T cells. Cells were stimulated at 37°C for 0, 1, 2, or 5 hours. After each time period, cells were placed on ice in the dark until all cells could be stained at the same time. Cells were washed with PBS containing 2% FBS and stained with CD8 mAb, Thy1.1 mAb, and a fluorescent cell viability stain (Live/Dead, Invitrogen) on ice. Cells were fixed and permeabilized with intracellular fixation and permeabilization buffer set from eBioscience. Intracellular staining was then completed by staining with IFN-γ, TNF-α and IL-2 mAbs on ice in the dark.

### Flow cytometry and mAbs

Spleens and tumors were harvested from mice after adoptive transfer. Spleens were passed through 70 *u*m cell strainers and lymphocytes were obtained after hypotonic lysis. Approximately 1 × 10^6^ were used for each staining. To determine the number of tumor-infiltrating lymphocytes (TILs), tumors were weighed and minced with a scalpel. The tumor was then digested in collagenase with DNase for 2 hours on a rotator. Small mononuclear cells within the tumor were enumerated by trypan blue exclusion, with approximately 1 × 10^6^ lymphocytes used for staining. TILs were calculated by determining the absolute number of CD8+ Thy1.1+ cells per milligram of tumor.

Fluorochrome conjugated Abs to CD4 (clone RM4-5), CD8 (clones 5H10 and 53-6.7), CD107a (clone 1D4B), IFN-γ (clone XMG1.2), TNF-α (clone MP6-XT22) and IL-2 (clone JES6-5H4) were obtained from BD Biosciences or Biolegend. Fluorochrome conjugated Abs to Thy1.1 (clone HIS51) and FoxP3 (clone FJK-16 s) were obtained from eBioscience. For intracellular cytokine staining, cells were washed with PBS containing 2% FBS and subsequently stained with surface markers. After extracellular staining, cells were fixed with Fixation Buffer (eBioscience) and permeabilized with Permeabilization Buffer (eBioscience). Intracellular staining was completed in Permeabilization Buffer on ice in the dark. Cells were stored at 4°C until analysis.

All FACS analysis was performed with the use of an LSRII (BD Biosciences). Gates were set based on samples stained with all fluorophores minus one. Only viable cells, as determined by negative staining with a dead cell stain (Live/Dead Fixable Near-IR Dead Cell Stain Kit, Invitrogen), were included in subsequent analyses. Data were analyzed using FlowJo software (Treestar).

### Assessment of serum cytokine levels

Serial blood measurements were obtained by retro-orbital sinus collection at the time points indicated. Collected blood was allowed to clot for 30 minutes before centrifugation for 10 minutes at 1000 × g. Serum was removed immediately and stored at -20°C until utilized. Cytokine serum levels were assayed with a Milliplex MAP Mouse Cytokine Magnetic Bead Panel (Millipore) and analysis was performed in the Center for AIDS Research ImmunoBioSpot Core Facility that is supported by National Institutes of Health awards CA-16042 and AI-28697, and by the UCLA AIDS Institute.

## Abbreviations

DC: Dendritic cell; TAA: Tumor-associated antigen; TBI: Total body irradiation; TIL: Tumor-infiltrating lymphocytes.

## Competing interests

The authors declare that they have no competing interests.

## Authors’ contributions

DNL, HS, LML, and RMP conceived and designed the experiments. DNL, HS, and RGE performed the experiments. DNL and HS analyzed the data. DNL and RMP wrote the manuscript. All authors read and approved the final manuscript.

## Supplementary Material

Additional file 1: Figure S1 Functional enhancement of Pmel T cell cytokine production. A. Restimulation of Pmel T cell splenocytes with cognate gp100_25-33_ peptide *ex vivo* for five hours. Representative plots indicate splenocytes restimulated 3 days following adoptive cell transfer and are gated from CD8+ Thy1.1+ Pmel T cells. Gates were set based on Pmel cells that lacked peptide stimulation for the 5 hour duration of the restimulation. Click here for file
