# Supplementary figures and images for: The histone deacetylase inhibitor, LBH589, promotes the systemic cytokine and effector responses of adoptively transferred CD8+ T cells
Source: J Immunother Cancer. 2014 Apr 15;2:8. doi: 10.1186/2051-1426-2-8 (PMC4105687; doi:10.1186/2051-1426-2-8)

Non-tumor bearing: day 3

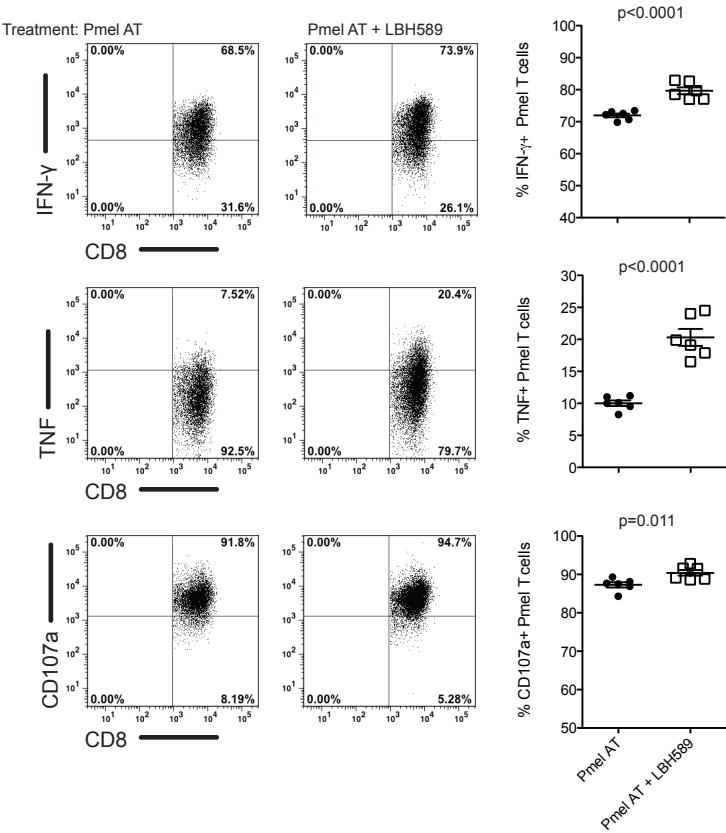

Supplement: Additional file 1: Figure S1 — Functional enhancement of Pmel T cell cytokine production. A. Restimulation of Pmel T cell splenocytes with cognate gp10025-33 peptide ex vivo for five hours. Representative plots indicate splenocytes restimulated 3 days following adoptive cell transfer and are gated from CD8+ Thy1.1+ Pmel T cells. Gates were set based on Pmel cells that lacked peptide stimulation for the 5 hour duration of the restimulation. [file 2051-1426-2-8-S1.pdf]
